# Supplementary material for: eNose-TB: A trial study protocol of electronic nose for tuberculosis screening in Indonesia
Source: PLoS One. 2021 Apr 21;16(4):e0249689. doi: 10.1371/journal.pone.0249689 (PMC8059810; doi:10.1371/journal.pone.0249689)
Supplement: S2 File — (DOCX) [file pone.0249689.s002.docx]

**PROTOKOL PENELITIAN**

**eNose TB: Inovasi Electronic-nose untuk Skrining Tuberkulosis di Indonesia**

**Kontributor**:

| **Nama** | **Institusi** | **Peran** | **email** |
| --- | --- | --- | --- |
| Yodi Mahendradhata | Universitas Gadjah Mada (UGM) | Principal investigator | ymahendradhata@ugm.ac.id |
| Riris Andono Ahmad | Universitas Gadjah Mada | Co-PI untuk epidemiologi | risandono_ahmad@ugm.ac.id |
| Ari Natalia Probandari | Universitas Sebelas Maret | Co-PI untuk implementasi riset | ari.probandari@gmail.com |
| Antonia Morita Iswari Saktiawati | Universitas Gadjah Mada | Studi klinis laboratoris | [a.morita@ugm.ac.id](mailto:a.morita_iswari@yahoo.com) |
| Bintari Dwihardiani | Universitas Gadjah Mada | Studi operasional | [bdwihardiani@gmail.com](mailto:bdwihardiani@gmail.com) |
| Kuwat Triyana | Faculty Fisika, UGM | Software dan hardware *e-nose* | triyana@ugm.ac.id |

**Sponsor** : Kementerian Riset, Teknologi dan Pendidikan Tinggi Republik Indonesia

**(dana)** Gedung D, Jalan Jenderal Sudirman Pintu Satu, Senayan, Jakarta Pusat 10270

**Kontak Peneliti:**

Nama : dr. Antonia Morita Iswari Saktiawati, PhD

Organisasi : Center for Tropical Medicine, Faculty of Medicine, Public Health and Nursing UGM

Email : [a.morita_iswari@yahoo.com](mailto:a.morita_iswari@yahoo.com) / [a.morita@ugm.ac.id](mailto:a.morita@ugm.ac.id)

Daftar Isi

[RINGKASAN 3](#_Toc55998481)

[BAB 1. PENDAHULUAN 4](#_Toc55998482)

[1.1. Latar Belakang 4](#_Toc55998483)

[1.2. Tujuan Penelitian 5](#_Toc55998484)

[BAB 2. METODE PENELITIAN 6](#_Toc55998485)

[2.1. Rancangan Penelitian 6](#_Toc55998486)

[2.2. Populasi Penelitian 8](#_Toc55998487)

[2.2.1. Populasi 8](#_Toc55998488)

[2.2.2. Sampel 8](#_Toc55998489)

[2.2.3. Kriteria Inklusi 8](#_Toc55998490)

[2.2.4. Kriteria Eksklusi 9](#_Toc55998491)

[2.4. Alat dan Bahan 9](#_Toc55998492)

[2.5. Prosedur Penelitian 10](#_Toc55998493)

[2.5.1. Pengumpulan sampel nafas 10](#_Toc55998494)

[2.5.2. Pengendalian Risiko dan Mitigasi Penularan Infeksi SARS-CoV2 11](#_Toc55998495)

[2.5.3. Pengumpulan data demografik dan klinis pasien 12](#_Toc55998496)

[2.5.4. Analisis 12](#_Toc55998497)

[REFERENSI 18](#_Toc55998498)

# RINGKASAN

Tuberkulosis (TB) adalah permasalahan global. Indonesia dengan jumlah populasi 264 juta, menjadi negara dengan beban TB terbesar ketiga. Kesenjangan antara jumlah kasus sebenarnya dengan kasus yang terdeteksi masih sangat tinggi. Skrining TB di Indonesia biasanya dilakukan dengan pemeriksaan gejala, namun pemeriksaan gejala hanya mempunyai sensitivitas sebesar 70%. Foto thorax dianjurkan sebagai alat skrining dengan sensitivitas 87%, namun foto thorax tidak praktis dibawa untuk penemuan kasus TB aktif dan tidak memungkinkan dibawa ke daerah terpencil.

Tes nafas dengan *electronic nose* berpotensi menjadi alat diagnosis. Tes nafas tidak invasif, dan sesuai digunakan untuk pasien yang kesulitan mengeluarkan dahak. *Electronic-noses* telah digunakan untuk diagnosis penyakit seperti asma, penyakit paru obstruktif kronis (PPOK), dan kanker. Universitas Gadjah Mada telah mengembangkan alat *electronic-nose* untuk mendiagnosis tuberkulosis. Alat tersebut mudah digunakan, portabel, hanya memerlukan listrik kecil untuk mengisi daya, dan dapat diproduksi dengan harga murah. Dengan bentuknya yang portabel, *electronic-nose* dapat dipakai untuk skrining TB. Pasien yang didiagnosis positif dengan *electronic-nose* kemudian dapat dirujuk untuk tes lanjutan yang lebih spesifik untuk konfirmasi diagnosis.

Penelitian ini bertujuan untuk meningkatkan penemuan kasus TB secara aktif di Indonesia dengan menggunakan *electronic-nose* sebagai alat skrining. Sebelum e-nose digunakan, peneliti melakukan training e-nose pada 27 pasien TB (sebagai kelompok kasus) dan 24 orang sehat (sebagai kelompok kontrol) di RS Respira, Yogyakarta. Kemudian dilanjutkan dengan fase pertama yaitu fase validasi e-nose dengan melibatkan 395 pasien suspek TB yang akan dilaksanakan di RSUP Surakarta, Puskesmas di kota Yogyakarta, dan Kulon Progo. Pada fase kedua akan melibatkan 1.383 responden, bersama dengan diluncurkannya penemuan kasus TB aktif dengan X-ray portabel di populasi berisiko tinggi di kota Yogyakarta dan Kabupaten Kulon Progo, *electronic nose* akan diujikan dan dibandingkan dengan X-ray dan gejala dalam penemuan kasus TB. Partisipan yang positif dengan *electronic-nose* atau radiologis, dan atau mempunyai gejala TB, akan dirujuk untuk tes cepat molekuler (Xpert MTB/Rif) yang direkomendasikan oleh WHO, yang mempunyai spesifisitas diagnosis lebih tinggi. Pada fase validasi dan fase skrining, referensi yang digunakan adalah Xpert MTB/Rif. Performa tes nafas sebagai skrining TB akan dibandingkan dengan performa gejala dan pemeriksaan radiologis dada. *Time and cost analysis* tes nafas sebagai skrining TB juga dilakukan. Data karakteristik partisipan akan dikumpulkan, seperti usia, berat badan, tinggi badan, jenis kelamin, kebiasaan merokok, kebiasaan minum alkohol, komorbiditas, komedikasi, pekerjaan, dan makanan dan minuman yang dikonsumsi sebelum tes nafas. Dengan kondisi Indonesia, di mana prevalensi TB masih tinggi, diharapkan *electronic-nose* dapat meningkatkan standar skrining TB.

*Keywords: tuberculosis, active case finding, pelacakan kontak, diagnosis, electronic-nose*

# BAB 1. PENDAHULUAN

## 1.1. Latar Belakang

Tuberkulosis adalah salah satu masalah kesehatan masyarakat global. Tuberkulosis merupakan penyakit infeksi yang menyebabkan kematian terbanyak di dunia akibat agen infeksi tunggal (1). Indonesia memegang ranking ketiga tertinggi untuk jumlah kasus Tuberkulosis di dunia, dan memiliki kesenjangan tinggi antara jumlah kasus sebenarnya dengan kasus yang terdeteksi (1).

Badan Kesehatan Dunia (WHO) merekomendasikan skrining untuk deteksi awal kasus Tuberkulosis sehingga dapat mengurangi transmisi dan meningkatkan kesembuhan pasien (2). Prioritas skrining dilakukan pada kontak erat Tuberkulosis (orang yang tinggal serumah maupun orang yang berada di ruangan yang sama dengan pasien Tuberkulosis selama satu periode tertentu), kemudian pada populasi dengan risiko Tuberkulosis (penderita HIV, diabetes, tinggal di lokasi dengan transmisi Tuberkulosis yang tinggi, *i.e.* daerah kumuh) (2). Skrining dengan gejala mempunyai sensitivitas 70% sedangkan skrining dengan foto thorax memiliki sensitivitas 87% (3), namun foto thorax memaparkan pasien pada radiasi dan tidak praktis untuk digunakan dalam penemuan kasus TB secara aktif dan di daerah terpencil. Dengan dampak klinis dan ekonomi TB yang signifikan, diperlukan pengembangan alat yang akurat untuk skrining, mudah digunakan, dan berbiaya rendah untuk digunakan di negara berpenghasilan bawah menengah seperti Indonesia.

Analisis napas menggunakan *electronic-nose (e-nose)* dapat digunakan sebagai diagnosis penyakit seperti asma (4) dan kanker paru (5). Pusat Kedokteran Tropis Universitas Gadjah Mada (UGM) bekerja sama dengan Fakultas Fisika UGM telah mengembangkan *e-nose* untuk mendeteksi tuberkulosis. *E-nose* tersebut praktis dibawa ke mana-mana, mudah digunakan, dan tanpa paparan radiasi sehingga sesuai sebagai alat skrining. Sekitar 30% kasus TB aktif saat ini tidak terdeteksi oleh pelayanan kesehatan di Indonesia (6).

Kami bertujuan mengadakan skrining TB dengan *e-nose* di Indonesia, di mana prevalensi TB masih tinggi di masyarakat (7), sehingga alat skrining yang mudah dibawa, mudah digunakan, dan murah akan sangat bermanfaat. Secara khusus, penelitian ini bertujuan untuk menginvestigasi potensi *e-nose* dibandingkan dengan pelacakan gejala dan radiologis dada yang saat ini dipakai sebagai standar skrining. Lebih lanjut, kami bertujuan untuk menganalisis waktu dan biaya dari algoritme skrining dengan *e-nose* untuk mendapatkan tambahan deteksi kasus; dan mengurangi kesenjangan deteksi kasus TB di Indonesia.

Urgensi penelitian terletak pada: pengembangan teknologi sebagai solusi untuk meningkatkan efektivitas program penyakit prioritas (tuberkulosis); memperbaiki penemuan kasus TB secara aktif sehingga meningkatkan kualitas pelayanan kesehatan; dan meningkatkan standar skrining TB. Sasaran utama penelitian ini adalah penduduk di Kota Yogyakarta dan Kabupaten Kulon Progo, Provinsi Daerah Istimewa Yogyakarta. Kota Yogyakarta dipilih karena merepresentasikan area dengan prevalensi TB yang tinggi dan Kabupaten Kulon Progo dipilih karena merepresentasikan area dengan banyak daerah terpencil dan sulit akses ke pusat kesehatan, dimana e-nose akan sangat bermanfaat dalam mendeteksi TB. Kota Yogyakarta mempunyai 18 Puskesmas dan 21 rumah-sakit (8) sedangkan Kabupaten Kulon Progo mempunyai 21 Puskesmas dan 9 rumah sakit (9) . Perkiraan insiden TB pada tahun 2019 di Kota Yogyakarta adalah 1.400 kasus, dan di Kabupaten Kulon Progo adalah 1.033 kasus (10). Pada tahun 2017, dua pertiga kasus TB terdeteksi di Kota Yogyakarta (10). Selain itu, Kota Surakarta juga menjadi sasaran penelitian ini karena pada tahun 2018, penemuan kasus TB di kota Surakarta mencapai sekitar 1.651 menurun dibanding tahun 2017 (11). Pada 2019, Kota Surakarta tercatat mempunyai 17 Puskesmas, 14 rumah sakit umum, dan 4 rumah sakit khusus (12).

## 1.2. Tujuan Penelitian

1.2.1. Menginvestigasi potensi tes nafas sebagai skrining TB.

1.2.2. Menganalisis waktu dan biaya dari algoritme skrining dengan e-nose untuk mendapatkan tambahan deteksi 1 kasus.

# BAB 2. METODE PENELITIAN

## 2.1. Rancangan Penelitian

Sebelum e-nose digunakan sebagai alat skrining, e-nose dilatih untuk mengenali pola nafas. Pada masa training alat, penelitian *case-control* telah dilakukan dengan melibatkan pasien TB sebanyak 27 orang diambil dari Rumah Sakit Respira, Yogyakarta, dan 24 orang sehat sebagai kontrol diambil dari lingkungan di sekitar pasien TB, untuk merepresentasikan populasi dengan kondisi sosial ekonomi yang sama. Sebagai bagian dari pemeriksaan rutin, semua partisipan dikategorikan dengan gejala klinis (batuk terus-menerus, penurunan BB >5% yang tidak disengaja, dan keringat malam), CXR, mikroskopis dahak, dan pemeriksaan Xpert MTB/Rif. Untuk tujuan penelitian, kami menambahkan biakan dahak/ kultur dahak, tes HIV, dan tes napas.

Pada fase pertama atau fase validasi, penelitian potong lintang akan dilakukan untuk memvalidasi mesin e-*nose* (validation phase). Penelitian akan dilakukan pada 395 pasien suspek TB di RSUP Surakarta, puskesmas di kota Yogyakarta dan kabupaten Kulon Progo. Sebagaimana pada tahap training alat, semua peserta penelitian pada fase validasi ditandai oleh gejala klinis (seperti batuk terus-menerus, penurunan berat badan >5% yang tidak disengaja, keringat malam), CXR, apus mikroskopis, pemeriksaan Xpert MTB/Rif sebagai referensi, dan tes nafas dengan *e-nose* melalui *sampling bag*.

Pada fase kedua atau fase skrining, penelitian potong lintang akan dilakukan di Kota Yogyakarta dan Kabupaten Kulon Progo yang akan melibatkan 1.383 responden. Bersamaan dengan diluncurkannya kegiatan penemuan kasus TB secara aktif, *e-nose* akan disandingkan dengan skrining gejala dan penggunaan radiologi dada (CXR) dalam kegiatan ini. Tim klinik mobil yang terdiri dari dokter, petugas radiologi, petugas laboratorium, dan perawat akan berkeliling ke tempat-tempat dengan risiko TB tinggi, yaitu: ruang tunggu rawat jalan Puskesmas dan rumah sakit, pemukiman kumuh, tempat kos, pesantren, dan penjara.

Partisipan akan diminta bernafas secara normal lewat *sampling bag* sampai kantung udara penuh dan ditanya apakah mengalami gejala TB, yaitu gejala utama TB (batuk berdahak >2 minggu, keringat malam tanpa aktifitas, penurunan berat badan tanpa penyebab jelas) dan gejala lain TB (batuk berdarah, demam hilang timbul >1 bulan, pembesaran kelenjar getah bening, sesak nafas dan nyeri dada), dan dilakukan pemeriksaan radiologis dada.

Pasien dengan hasil radiologis dan atau tes nafas positif dan atau batuk lebih dari dua minggu atau batuk darah atau gejala TB ekstra paru akan dirujuk untuk konfirmasi diagnosis menggunakan tes yang direkomendasikan oleh WHO, yang mempunyai spesifisitas lebih tinggi, yaitu Xpert MTB/Rif. Performa tes nafas sebagai skrining TB akan dibandingkan dengan performa gejala dan atau radiologis dada. Xpert MTB/Rif dipakai sebagai referensi karena penggunaannya yang sudah luas, meminimalisir mobilitas sputum, dan memberikan hasil yang cepat (dalam waktu 2 jam). Data demografik dan klinis partisipan akan dikumpulkan. Waktu dan biaya dari algoritme skrining dengan *e-nose* untuk mendapatkan tambahan deteksi 1 kasus TB akan dianalisis.

**Gambar 1**. Alur skrining pasien dengan *e-nose* dan pelacakan gejala

Klinik mobil pelacakan TB aktif di ruang tunggu pasien rawat jalan Puskesmas, RS, pemukiman, kumuh, pesantren, dan tempat kos. Semua partisipan menjalani skrining dengan CXR portabel, e-nose, dan gejala klinis

Partisipan dengan tes nafas (-) DAN batuk >2 minggu atau batuk darah (-) DAN gejala TB ekstra paru (-) DAN radiologis (-)

Partisipan dengan CXR abnormal ATAU tes nafas (+) ATAU batuk 2 minggu atau batuk darah (+) ATAU gejala TB ekstra paru (+)

Dirujuk untuk pemeriksaan Xpert MTB/Rif dahak untuk konfirmasi diagnosis

1. Analisis performa tes nafas sebagai skrining TB dibandingkan dengan performa pelacakan gejala atau radiologis dada
2. *Time and cost analysis* dari tes nafas sebagai skrining TB

## 2.2. Populasi Penelitian

### 2.2.1. Populasi

Pada fase validasi adalah pasien anak maupun dewasa yang datang ke RSUP Surakarta, dan Puskesmas di kota Yogyakarta dan kabupaten Kulon Progo. Pada fase skrining, populasi adalah penduduk di Kota Yogyakarta dan Kabupaten Kulon Progo, Provinsi DIY baik anak maupun dewasa.

### 2.2.2. Sampel

- Tahap Training Alat

Tahap training e-nose dilakukan pada 27 orang pasien TB dan 24 orang sehat sebagai kontrol negatif. Berdasarkan jurnal dari Bruins et al dan Zetola NM (13, 14), jumlah sampel ini dapat diterima oleh mesin *e-nose* untuk dapat mengenali pola napas.

- Fase Validasi

Minimum sampel yang dibutuhkan untuk fase validasi dihitung menggunakan rumus n=$\frac{{Z_{\propto/2}}^{2}xSNx(1-SN)}{d^{2}}/P$ (13)

dengan $Z_{\propto/2}$ = 1.96, nilai SN 90%, nilai d 5%, P value 35%, sehingga jumlah minimal responden yang dibutuhkan sebanyak 395 responden.

- Fase Skrining

Jumlah sampel pada fase skrining dihitung dengan rumus sama dengan fase validasi dengan nilai $Z_{\propto/2}$1.96, nilai SN 90%, nilai d 5%, P value 10%, sehingga jumlah minimum responden yang dibutuhkan sebanyak 1.383 responden.

### 2.2.3. Kriteria Inklusi

Fase Validasi:

- Anak dan dewasa
- Bersedia berpartisipasi (penandatanganan *informed consent*)*.*
- Dapat menghasilkan sampel untuk pemeriksaan Xpert MTB/Rif
- Dapat menghasilkan sampel nafas

Fase Skrining:

- Anak dan dewasa
- Berdomisili di kota Yogyakarta dan kabupaten Kulon Progo.
- Bersedia berpartisipasi (penandatanganan *informed consent*)*.*
- Tidak sedang dalam pengobatan TB.

### 2.2.4. Kriteria Eksklusi

- Tidak mampu bernafas secara normal selama 2 menit dikarenakan penyakit pernafasan.
- Tidak mempunyai hasil pemeriksaan penunjang
- Pengukuran tes nafas tidak valid

## 2.3. Pembutaan/ *Blinding*

Untuk menjamin validitas penelitian, penelitian ini menggunakan *triple blind masking*, dimana subjek penelitian tidak mengetahui hasil interpretasi sampel nafas. Pengambil sampel nafas terbutakan terhadap hasil pemeriksaan sampel laboratorium, dan pemeriksa sampel laboratorium tidak mengetahui hasil dari setiap pengambilan sampel nafas yang telah dilakukan. Pengolah data akhir juga tidak dapat melihat hasil Xpert atau pemeriksaan penunjang lain. Data pengambilan sampel nafas disimpan dalam bentuk grafik yang interpretasinya akan dilakukan oleh pengolah data pada tahap akhir.

## 2.4. Alat dan Bahan

Sistem *e-nose* secara keseluruhan terdiri dari sistem *sampling* (1 – 3), sistem larik sensor (4), dan sistem akuisisi data (5 dan 6) seperti pada gambar berikut:


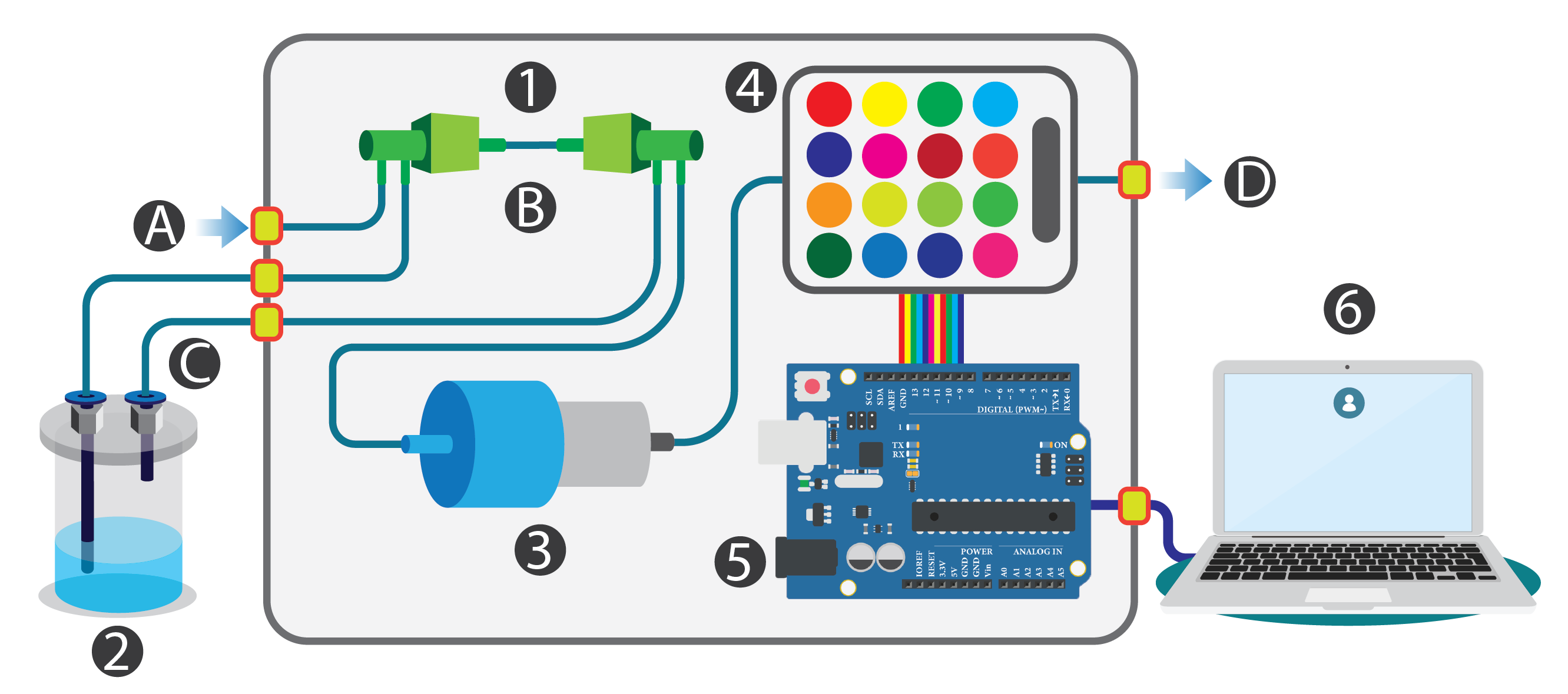


Sedangkan sistem perangkat lunak *E-nose* terdiri dari dua bagian pemrograman, yaitu pemrograman mikrokontroler (Arduino MEGA 2560) dan pemrograman perangkat lunak *data logger* (Windows).

Partisipan bernafas melalui *sampling bag* sekali pakai sampai kantong udara penuh. Sampling bag dihubungkan dengan HEPA-filter untuk melindungi *electronic-nose* terkontaminasi bakteri dan virus. Untuk mencegah penularan COVID-19, kami melakukan beberapa pencegahan seperti pengadaan bahan habis pakai yaitu masker N95, masker bedah, *face shield, hand schoon, hand sanitizer,* alkohol 70%, dan kotak sampah medis.

## 2.5. Prosedur Penelitian

### 2.5.1. Pengumpulan sampel nafas

Sebelum proses pengambilan sampel nafas, dokter akan memeriksa pasien dan gejala tuberkulosis. Setelah dokter mengkonfirmasi bahwa pasien memenuhi kriteria inklusi dan eksklusi, petugas DOTS akan memberikan penjelasan terkait penelitian, dan meminta *informed consent* jika pasien bersedia. Petugas memberikan *sampling bag/* kantong pengumpul sampel nafas dengan menutup lubang selang oksigen. Petugas akan memberikan arahan pengumpulan sampel nafas ke dalam kantong. Petugas akan tetap mendampingi pasien saat pengumpulan sampel nafas pada bilik/ tempat khusus berdahak dengan berjarak 1 meter. Cara pengambilan sampel nafas yaitu partisipan akan diminta melakukan nafas dalam dengan tetap memakai masker sebanyak 2 kali nafas, kemudian saat nafas ketiga, partisipan diminta menarik nafas dalam dan menghembuskan nafas secara *forced expiratory volume* ke dalam kantong pengumpul nafas sekali pakai sampai kantong sampel nafas penuh kemudian diikat bagian atasnya agar udara dalam kantong tidak keluar. Setelah kantong pengumpul nafas penuh dan diikat, kantong dimasukkan ke dalam kotak tertutup dan diserahkan kepada petugas. Pasien akan mengumpulkan tes nafas sebanyak dua kali.

*Sampling bag/* kantong pengumpul nafas yang telah berisi nafas partisipan kemudian dihubungkan ke HEPA-filter dengan konektor pada satu sisi dan sisi yang lain dihubungkan ke mesin e-nose. Setelah menggunakan *e-nose*, petugas akan melakukan disinfeksi peralatan baik mesin *e-nose*, selang penghubung, dan benda-benda disekitar menggunakan alkohol 70% kemudian ditunggu sampai kering dan *e-nose* siap digunakan untuk partisipan berikutnya. *Sampling bag*, konektor, dan HEPA-filter yang telah dipakai kemudian dimasukkan ke dalam kotak sampah medis.

Petugas ataupun tenaga medis yang bertugas mengoperasikan mesin e-nose dan mengambil sampel akan mendapatkan pelatihan terlebih dahulu mengenai gambaran penelitian, cara mengoperasikan mesin e-nose serta pelatihan penggunaan APD sesuai dengan SOP oleh peneliti. Protokol yang akan dilakukan sebagai antisipasi penularan COVID-19 dari pengambilan sampel sebagai berikut:

1. Mesin e-nose tidak akan dikeluarkan dari ruang lab sampai benar-benar selesai dan akan dibungkus dengan wrap plastik
2. Apabila mesin dapat dihubungkan dengan koneksi internet, sehingga data akses secara elektronik menggunakan jaringan internet, maka mesin tidak perlu dikeluarkan.
3. Jika peneliti diperlukan untuk masuk, maka peneliti wajib mengikuti protocol kesehatan yang berlaku dan menggunakan APD level 3 sesuai prosedur medis
4. Penggunaan kantong pengumpul sampel nafas desain khusus untuk pengambilan sampel nafas dan penggunaan tunggal, setelah selesai akan dibuang di limbah medis infeksius sesuai dengan protocol kesehatan
5. Kantong pengumpul sampel nafas yang telah penuh diikat dan dimasukkan dalam kotak tertutup kedap udara untuk meminimalisir transmisi sebelum kantong sampel dihubungkan ke mesin e-nose.

Efek samping dari tindakan pengambilan sampel nafas secara *forced expiratory volume* sebanyak 3 kali pada umumnya jarang terjadi. Studi melaporkan bahwa efek samping paling sering adalah rasa tidak nyaman berupa mual, muntah, dan rasa berkunang-kunang apabila dilakukan lebih dari 10x secara berurutan. Oleh karena itu, antisipasi yang dilakukan dalam penelitian ini adalah pengambilan nafas maksimal 3 kali hembusan setiap kali pengambilan. Jika terjadi efek samping, perawat telah dilatih untuk memberikan pertoolongan yaitu dengan memposisikan pasien dalam *tredelenberg* di mana pasien ditidurkan dan kaki ditinggikan, kemudian pasien diberikan tambahan suplementasi oksigen dengan nasal kanul. Evaluasi keluhan dilakukan 15 menit kemudian dan dilaporkan pada dokter penanggungjawab dan peneliti.

### 2.5.2. Pengendalian Risiko dan Mitigasi Penularan Infeksi SARS-CoV2

Dalam rangka mencegah penularan COVID-19 selama penelitian berlangsung, peneliti telah membuat SOP kerja yang menerapkan protokol kesehatan dan penggunaan APD yang sesuai dengan pedoan pencegahan dan pengendalian COVID-19 revisi ke-5 oleh Kementerian Kesehatan serta sesuai dengan petunjuk praktek dan SOP RSUP Surakarta dan Puskesmas.

Proses penjelasan kepada partisipan, pengisian *informed consent* serta *Case Report Form* (CRF) dilakukan oleh perawat yang terlatih degan menggunakan standar APD level 2 (masker bedah, gown, sarung tangan, face shield/ google). Petugas pendamping pengumpulan sampel nafas partisipan dan petugas yang mengoperasikan mesin e-nose menggunakan standar APD level 3 (masker N95 berlapis masker bedah, gown, face shield/ goggles, sarung tangan bedah karet, penutup kepala, sepatu pelindung).

Sampah medis berupa kantong pengumpul sampel nafas, konektor, HEPA-filter, tissue/ kapas alcohol, sarung tangan bedah karet, masker bedah/ N95 dimasukkan dalam tempat sampah kategori limbah medis infeksius. Untuk selanjutnya proses dekontaminasi dan penghancuran diserahkan kepada PPI dan protokol pengolahan limbah medis di RSUP Surakarta dan Puskesmas.

### 2.5.3. Pengumpulan data demografik dan klinis pasien

Usia, berat badan, tinggi badan, jenis kelamin, kebiasaan merokok, kebiasaan minum alkohol, komorbiditas (diabetes, asma, PPOK, HIV, flu, bronkitis, bronkiektasis, fibrosis paru, abses paru, empyema, penyakit paru polikistik), komedikasi (obat inhalasi dan antibiotik), pekerjaan, makanan dan minuman yang dikonsumsi sebelum tes nafas. Dua peneliti akan memasukkan data dan memastikan bahwa tidak ada data yang hilang atau kesalahan pengisian data.

### 2.5.4. Analisis

**Interpretasi data e-nose**

Interpretasi data e-nose menggunakan perangkat lunak analisis data multivariate (kemometrik) yang menggunakan bahasa pemrograman *open source* R versi 3.5.1 dan Python versi 3.7. Bahasa pemrograman R adalah bahasa pemrograman yang ditujukan untuk komputasi statistik (seperti *linear* dan *nonlinear modelling*, *classical statistical tests*, *time-series analysis*, *classification*, dan *clustering*) dan pengolahan grafik. R dalam penelitian ini digunakan sebagai mesin pengolah data yang terdiri dari fungsi ekstraksi ciri dan fungsi analisis data multivariat. GUI perangkat lunak analisis data multivariat dibangun dengan menggunakan Microsoft Visual Studio 2019.

Satu siklus pengambilan data terdiri dari fase *delay*, fase *sampling* dan fase *purging*. Untuk satu kali pengambilan data diperoleh data yang besar dan kemungkinan banyak data *redundant* (laju data 10 larik data per detik), sehingga prosedur ekstraksi ciri diaplikasikan untuk memperoleh informasi penting dari suatu respons sensor. Dalam penelitian ini, beberapa metode ekstraksi ciri digunakan dan dibandingkan untuk memperoleh hasil akurasi terbaik. Untuk data *E-tongue*, metode ekstraksi ciri yang dilakukan hanya dengan mengambil nilai rata-rata data *sampling* untuk masing-masing sensor.

Melalui perintah RUN, program akan melakukan prosedur ekstraksi ciri semua berkas data (format *.csv) yang berada di dalam folder terpilih. Dalam penelitian ini, 16 metode ekstraksi ciri digunakan, di mana *V_i,j_* adalah data ke-*i* (10 data setiap detik pengambilan data) untuk sensor-*j*. Dalam pemrograman R, analisis PCA dilakukan dengan sebuah fungsi “*prcomp*” dalam paket *library base*, sedangkan analisis t-SNE dilakukan dengan fungsi “*Rtsne*” dalam paket *library* *Rtsne*. LDA digunakan untuk melihat sebaran data yang terklasifikasi menggunakan model pembelajaran LDA (*supervised learning*). Prosedur LDA dalam penelitian ini menggunakan fungsi “*lda*” di dalam paket *library MASS*. Simulated Annealing digunakan sebagai metode seleksi fitur, mencari fitur-fitur terpenting dan menghilangkan fitur yang redundan, menggunakan algoritma yang meniru cara kerja proses pendinginan logam. *Simulated Annealing* dalam bahasa R dikombinasikan dengan LDA sebagai model pembelajarannya melalui paket *library subselect.*

Classification dan Regression merupakan program utama dalam analisis data kemometrik. Kedua model tersebut dibangun dengan menggunakan paket *library caret* (*Classification And REgression Training*) yang menyediakan fungsi-fungsi penting di dalam analisis data kemometrik, seperti *data splitting*, *pre-processing*, *feature selection*, model *training* dan *tuning*, *variable importance estimation,* dll. ROC digunakan untuk melakukan analisis *Receiver operating characteristic* (ROC) terhadap 2 kelas label. *Library caret* juga digunakan dalam prosedur analisis ROC. Radar Plot digunakan untuk menampilkan variabilitas data rata-rata tiap kelas label dengan tujuan sebagai hipotesis awal ada perbedaan respons sensor terhadap kelas label yang berbeda.

Bahasa pemrograman Python juga digunakan di dalam prosedur analisis data kemometrik. Prosedur analisis data kemometrik di lingkungan Python membutuhkan beberapa paket pendukung, seperti *Numpy*, *pandas*, *sklearn*, *TensorFlow*, dan *Keras. Anaconda* digunakan di dalam penelitian ini sebagai platform *data science* yang menyediakan *library machine learning* dan bekerja di Python versi terbaru. Dalam penelitian ini, TORCLIB.py dikembangkan dan dibangun dengan berbagai *library* khususnya untuk pengolahan analisis data kemometrik.

**Analisis statistik**

Pada fase validasi, sensitivitas, spesifisitas, *positive dan negative predictive values* (PPV dan NPV) dari tes nafas, menggunakan Xpert MTB/Rif sebagai standar referensi. Pada setiap variabel (seperti usia, BMI), kurva ROC satu stratum yang menunjukkan sensitivitas dan spesifisitas tes napas, akan dibandingkan dengan kurva ROC stratum lain. Hubungan antara variabel tes nafas dan sensitivitas-spesifisitas ditunjukkan dengan perbedaan yang signifikan dari AUC antar strata (p<0.05).

Pada fase skrining, performa skrining dengan tes napas akan dibandingkan dengan skrining gejala atau pemeriksaan rontgen dengan menghitung kesepakatan positif dan negatif *(positive and negative agreements)* antara tes nafas dan gejala klinis atau pemeriksaan rontgen. Waktu dan biaya dari algoritme skrining dengan e-nose untuk mendapatkan tambahan deteksi 1 kasus TB akan dihitung dengan waktu rata-rata yang diperlukan dan biaya rata-rata yang dihabiskan dari awal skrining hingga penemuan kasus TB. Analisis statistik akan dilakukan dengan STATA/SE 15 (Lisensi: Universitas Gadjah Mada).

**BAB 3. PERTIMBANGAN ETIK**

Penelitian akan dilakukan sesuai dengan prinsip Deklarasi Helsinksi 2013 dan Cara Uji Klinik yang Baik, dan dengan persetujuan Komite Etik, Fakultas Kedokteran, Kesehatan Masyarakat, dan Keperawatan, Universitas Gadjah Mada, Yogyakarta, Indonesia. Pada penelitian tahun pertama (tahap training alat), sudah mendapatkan persetujuan komite etik dengan nomor KE/FK/0769/EC/2019 pada tanggal 5 Juli 2019 yang berlaku selama satu tahun.

Pada fase validasi, dokter penanggungjawab pasien di RSUP Surakarta dan Puskesmas akan memberikan informasi kepada pasien yang suspek TB mengenai penelitian ini, dan menawarkan pasien untuk bertemu salah satu tim peneliti atau petugas di RSUP Surakarta yang dilatih untuk memberikan informasi mengenai penelitian. Tim peneliti atau petugas akan memberikan informasi mengenai penelitian ini secara lisan dan tertulis. Bila pasien bersedia, pasien akan menandatangani *informed-consent*. Apabila pasien adalah anak-anak berusia di bawah 12 tahun, *informed consent* dimintakan ke orang tua. Untuk anak remaja usia 12-18 tahun, dimintakan *accent informed consent* di mana anak turut memberikan persetujuan terhadap rencana keikutsertaan terhadap penelitian.

Pada penelitian fase skrining, perawat/ petugas dalam tim klinik mobil akan memberikan informasi kepada calon partisipan mengenai penelitian ini. Bila subyek bersedia, subyek penelitian akan menandatangani *informed-consent*.

Partisipan akan diberikan penjelasan terkait kemungkinan efek samping dari proses pengambilan sampel nafas apabila dilakukan secara berlebihan seperti terjadinya reflex vagal berupa sensasi pusing, mual, berputar. Apabila ini terjadi, partisipan akan ditolong dengan memposisikan pasien ke dalam posisi *tredelenburg* dengan posisi kaki ditinggikan dari kepala. Partisipan tidak akan dibayar untuk berkontribusi dalam penelitian ini, namun bila ada biaya yang dikeluarkan partisipan untuk berpartisipasi dalam penelitian ini, biaya tersebut akan ditukar.

Tim peneliti akan menyimpan semua data di tempat yang aman, dan hanya peneliti yang mempunyai akses ke data ini. Data dapat dibuka kepada otoritas legal dan petugas monitoring dan audit. Dalam penelitian ini, tidak ada data sampel apapun yang akan dibawa ke luar negeri dan digunakan untuk apapun terkait pengembangan alat di luar negeri.

Dokter yang tidak terlibat dalam penelitian (*independent physician*) disediakan untuk menjawab pertanyaan dari calon partisipan, jika mereka membutuhkan. Ada satu dokter penanggung jawab klinis di masing-masing rumah sakit/tempat penelitian yang terlibat dalam penelitian ini.

**BAB 4. TIMELINE PENELITIAN**

Fase Validasi

| No | Nama Kegiatan | Bulan ke- | | | | | | | |
| --- | --- | --- | --- | --- | --- | --- | --- | --- | --- |
|  |  | 1 (Sept. 2020) | 2 (Okt. 2020) | 3 (Nov. 2020) | 4 (Dec. 2020) | 5 (Jan. 2021) | 6 (Feb. 2021) | 7 (March 2021) | 8 (April 2021) |
| 1. | Pengurusan ethical clearance | x | x |  |  |  |  |  |  |
| 2. | Penjelasan mengenai penelitian kepada RSUP Surakarta | x | x |  |  |  |  |  |  |
| 3. | Pelatihan penggunaan electronic-nose dan simulasi alur penelitian |  | x |  |  |  |  |  |  |
| 4. | Perekrutan pasien untuk fase validasi *electronic-nose* |  |  | x | x | x | x | x | x |
| 5. | Analisis data |  |  |  |  |  |  |  |  |
| 6. | Penulisan manuscript dan publikasi | x | x | x |  |  |  |  |  |

Fase Skrining

| No | Nama Kegiatan | Bulan ke- | | | | | | | | | | | |
| --- | --- | --- | --- | --- | --- | --- | --- | --- | --- | --- | --- | --- | --- |
|  |  | 1 | 2 | 3 | 4 | 5 | 6 | 7 | 8 | 9 | 10 | 11 | 12 |
| 1. | Penjelasan mengenai penelitian kepada Dinas Kesehatan Provinsi DIY, Dinas Kesehatan dan Puskesmas di Kota Yogyakarta dan Kab. Kulon Progo | x |  |  |  |  |  |  |  |  |  |  |  |
| 2. | Pelatihan penggunaan electronic-nose dan simulasi alur penelitian |  | x |  |  |  |  |  |  |  |  |  |  |
| 3. | Skrining TB dengan electronic-nose |  |  | x | x | x | x | x | x | x |  |  |  |
| 4. | Analisis data |  |  |  |  |  |  |  |  |  | x |  |  |
| 5. | Penulisan manuskrip dan publikasi |  |  |  |  |  |  |  |  |  |  | x | x |

# REFERENSI

1. World Health Organization. Global Tuberculosis Report 2019 [Internet]. Geneva; [cited 2019 Nov 27]. Available from: https://apps.who.int/iris/bitstream/handle/10665/329368/9789241565714-eng.pdf

2. World Health Organization. Systematic Screening for Active Tuberculosis: Principles and Recommendations. Geneva; 2015. Available from: https://www.who.int/tb/tbscreening/en/

3. van’t Hoog AH, Langendam MW, Mitchell E, Cobelens FG, Sinclair D, Leeflang MMG, et al. A systematic review of the sensitivity and specificity of symptom- and chest-radiography screening for active pulmonary tuberculosis in HIV-negative persons and persons with unknown HIV status. In: World Health Organization, editor. WHO/HTM/TB. Geneva: World Health Organization; 2013. p. 44–7. (Systematic screening for active tuberculosis: principles and recommendations).

4. Fens N, Zwinderman AH, van der Schee MP, de Nijs SB, Dijkers E, Roldaan AC, et al. Exhaled breath profiling enables discrimination of chronic obstructive pulmonary disease and asthma. Am J Respir Crit Care Med. 2009 Dec;180(11):1076–82.

5. Dragonieri S, Annema JT, Schot R, van der Schee MP, Spanevello A, Carratu P, et al. An electronic nose in the discrimination of patients with non-small cell lung cancer and COPD. Lung Cancer. 2009 May;64(2):166–70.

6. Riono P. TB Elimination in Indonesia [Internet]. 2018 [cited 2018 Nov 13]. Available from: http://www.depkes.go.id/resources/download/info-terkini/materi pra rakerkesnas 2018/Pakar TBC.pdf

7. World Health Organization. Health System Profile of Indonesia [Internet]. 2013 [cited 2016 Mar 20]. Available from: http://www.ino.searo.who.int/en/Section3_24.htm

8. Dinas Kesehatan Kota Yogyakarta. Profil Kesehatan Tahun 2019 Kota Yogyakarta. Yogyakarta; 2020. Available from: https://kesehatan.jogjakota.go.id/uploads/dokumen/profil_dinkes_2019_data_2018.pdf

9. Dinas Kesehatan Kabupaten Kulon Progo. Profil Kesehatan Tahun 2020. Yogyakarta; 2020. Available from: https://dinkes.kulonprogokab.go.id/detil/726/profil-kesehatan

10. Dinas Kesehatan Daerah Istimewa Yogyakarta. Profil Kesehatan Daerah Istimewa Yogyakarta Tahun 2019. Yogyakarta; 2020. Available from: https://www.dinkes.jogjaprov.go.id

11. Kementerian Kesehatan Republik Indonesia. Peran Pemerintah Daerah dalam Menurunkan Prevalensi Tuberkulosis Kota Surakarta [Internet]. Jakarta; 2019 [cited 2020 Oct 1]. Available from: https://www.kemkes.go.id/resources/download/info-terkini/rakerkesnas-2019/SESI II/Kelompok 5/2-Peran-Pemerintah-Daerah-dalam-Menurunkan-Prevalensi-TB.pdf

12. Badan Pusat Statistik. Badan Pusat Statistik Kota Surakarta [Internet]. 2020 [cited 2020 Oct 1]. Available from: https://surakartakota.bps.go.id/statictable/2020/04/01/178/jumlah-rumah-sakit-umum-rumah-sakit-khusus-rumah-sakit-bersalin-rumah-bersalin-puskesmas-klinik-balai-kesehatan-posyandu-dan-polindes-menurut-kecamatan-di-kota-surakarta-2018-dan-2019.html

13. Bruins M, Rahim Z, Bos A, Van De Sande WWJ, Endtz HP, Van Belkum A. Diagnosis of active tuberculosis by e-nose analysis of exhaled air. Tuberculosis [Internet]. 2013 Mar [cited 2020 Oct 26];93(2):232–8. Available from: https://pubmed.ncbi.nlm.nih.gov/23127779/

14. Zetola NM, Modongo C, Matsiri O, Tamuhla T, Mbongwe B, Matlhagela K, et al. Diagnosis of pulmonary tuberculosis and assessment of treatment response through analyses of volatile compound patterns in exhaled breath samples. J Infect [Internet]. 2017 Apr 1 [cited 2020 Oct 26];74(4):367–76. Available from: https://pubmed.ncbi.nlm.nih.gov/28017825/

15. Fenn Buderer NM. Statistical methodology: I. Incorporating the prevalence of disease into the sample size calculation for sensitivity and specificity. Acad Emerg Med [Internet]. 1996 [cited 2020 Oct 26];3(9):895–900. Available from: https://pubmed.ncbi.nlm.nih.gov/8870764/
