# Supplementary material for: eNose-TB: A trial study protocol of electronic nose for tuberculosis screening in Indonesia
Source: PLoS One. 2021 Apr 21;16(4):e0249689. doi: 10.1371/journal.pone.0249689 (PMC8059810; doi:10.1371/journal.pone.0249689)
Supplement: S3 File — (DOCX) [file pone.0249689.s003.docx]

**STUDY PROTOCOL**

**eNose TB: Electronic-nose Innovation for Tuberculosis Screening in Indonesia**

**Protocol contributors**:

| **Name** | **Institution** | **Roles** | **email** |
| --- | --- | --- | --- |
| Yodi Mahendradhata | Universitas Gadjah Mada (UGM) | Principal investigator | ymahendradhata@ugm.ac.id |
| Riris Andono Ahmad | Universitas Gadjah Mada | Co-PI for epidemiology | risandono_ahmad@ugm.ac.id |
| Ari Natalia Probandari | Universitas Sebelas Maret | Co-PI for research implementation | ari.probandari@gmail.com |
| Antonia Morita Iswari Saktiawati | Universitas Gadjah Mada | Clinical laboratory study | [a.morita@ugm.ac.id](mailto:a.morita_iswari@yahoo.com) |
| Bintari Dwihardiani | Universitas Gadjah Mada | Operational study | [bdwihardiani@gmail.com](mailto:bdwihardiani@gmail.com) |
| Kuwat Triyana | Faculty of Physics, UGM | Software and hardware of the *e-nose* | triyana@ugm.ac.id |

**Sponsor** : Ministry of Research, Technology and Higher Education of Republic Indonesia

**(funding)** Gedung D, Jalan Jenderal Sudirman Pintu Satu, Senayan, Jakarta Pusat 10270

**Reseacher Contact**

Name : dr. Antonia Morita Iswari Saktiawati, PhD

Organization : Center for Tropical Medicine, Faculty of Medicine, Public Health and Nursing UGM

Email : [a.morita_iswari@yahoo.com](mailto:a.morita_iswari@yahoo.com) / [a.morita@ugm.ac.id](mailto:a.morita@ugm.ac.id)

Table of content

[ABSTRACT 3](#_Toc59197099)

[CHAPTER 1. INTRODUCTION 4](#_Toc59197100)

[1.1. Background 4](#_Toc59197101)

[1.2. Research Objectives 5](#_Toc59197102)

[CHAPTER 2. RESEARCH METHODOLOGY 6](#_Toc59197103)

[2.1. Research Design 6](#_Toc59197104)

[2.2. Research Population 8](#_Toc59197105)

[2.2.1. Population 8](#_Toc59197106)

[2.2.2. Sample 8](#_Toc59197107)

[2.2.3. Inclusion Criteria 8](#_Toc59197108)

[2.2.4. Exclusion Criteria 9](#_Toc59197109)

[2.3. Blinding 9](#_Toc59197110)

[2.4. Tools and Materials 9](#_Toc59197111)

[2.5. Research Procedure 10](#_Toc59197112)

[2.5.1. Collecting of the exhaled breath sample 10](#_Toc59197113)

[2.5.2. Risk Control and Mitigation of SARS-CoV Infection Transmission 11](#_Toc59197114)

[2.5.3. Collection of patient clinical and demographic data 12](#_Toc59197115)

[2.5.4. Analysis 12](#_Toc59197116)

[REFERENCES 16](#_Toc59197117)

# ABSTRACT

Tuberculosis (TB) is a global public health problem. Indonesia, with a population of 264 million, is the country with the third-largest burden of TB. There is a high gap between the estimated number of incident cases and the notification of new cases. Screening for TB in Indonesia is usually carried out by symptoms examination, but symptom examination only has a sensitivity of 70%. Chest X-ray is recommended as a screening tool with a sensitivity of 87%, but chest X-ray is not practical to carry for TB's active case finding and is impossible to be carried to the remote areas.

A breath test with an electronic has the potency to be a diagnostic tool. The breath test is non-invasive and suitable for patients who have difficulty expelling sputum. Electronic-noses have been used to diagnose asthma, chronic obstructive pulmonary disease (COPD), and cancer. Universitas Gadjah Mada has developed an electronic-nose device for diagnosing tuberculosis. It is easy-to-use, portable, requires only a small of electricity to operate, and can be produced at a low price. With its portable form, the electronic-nose can be used for tuberculosis screening. Patients diagnosed positive by the electronic-nose can be referred for more specific follow-up tests to confirm the diagnosis.

This study aims to increase TB's active case finding in Indonesia by using the electronic-nose as a screening tool. Before the e-nose was used, the investigators conducted electronic-nose training on 27 TB patients and 24 healthy people as controls in Respira Lung Hospital, Yogyakarta. Afterward, it will be continued with the first phase (validation phase), involving 395 presumptive TB patients in Surakarta General Hospital, and Puskesmas (primary health centers) in the municipality of Yogyakarta and Kulon Progo District. In the second phase (screening phase), 1,383 respondents will be involved. Along with the launch of the active TB cases findings with portable X-rays in high-risk populations in the municipality of Yogyakarta and Kulon Progo District, electronic noses will be paired with x-rays and symptoms examination in the active TB case findings. Participants who are positive with electronic-nose or radiology or have TB symptoms will be referred for a rapid molecular test (Xpert MTB / Rif) recommended by WHO, which has a higher diagnostic specificity. In the validation and screening phases, the Xpert MTB / Rif is used as a reference. The breath test's performance as a tuberculosis screening will be compared with the performance of symptoms and chest X-ray examinations. Time and cost analysis of breath tests as tuberculosis screening are also calculated. Data on participant characteristics will be collected, such as age, weight, height, sex, smoking habits, alcohol consumption, comorbidities, comedication, occupation, and food and drink consumed before the breath test. As Indonesia has a high prevalence of TB, it is expected that electronic-nose can increase the standard of TB screening.

*Keywords: tuberculosis, active case finding, contact tracing, diagnosis, electronic-nose*

# CHAPTER 1. INTRODUCTION

## 1.1. Background

Tuberculosis is a global public health problem. Tuberculosis is an infectious disease that causes the highest death in the world due to a single infectious agent (1). Indonesia ranks as third-highest TB burdened country in the world and carries a high gap between the estimated number of incident cases and the notifications of new cases (1).

World Health Organization (WHO) recommends screening for early detection of TB cases to reduce transmission and improve patient outcomes (2). The screening is prioritized for people who have close contacts with TB (who live in the same household or frequent contact with the sputum smear-positive TB patients), and afterward for populations at risk of TB (people with HIV, diabetes, residents of the area with high TB transmission, *i.e.,*slum areas) (2). Screening with clinical symptoms has a sensitivity of 70%, while screening with chest radiography has a sensitivity of 87% (3). However, chest radiography exposes patients to radiation and is not practical to use in remote areas. Due to TB’s significant clinical and economic impact, it is of great importance to developing screening tools that are accurate, easy to use, and produced at a low cost, thus can be widely used in lower-middle-income countries such as Indonesia.

Breath analysis using an electronic-nose (e-nose) can be used to diagnose diseases such as asthma (4) and lung cancer (5). Center for Tropical Medicine, Universitas Gadjah Mada (UGM), in collaboration with the Faculty of Physics UGM, has developed an e-nose to detect tuberculosis. The e-nose is practice to carry, easy to use, and without radiation exposure making it suitable as a screening tool. Approximately 30% of active TB cases are currently undetected by Indonesia’s health services (6).

We aim to conduct TB screening with an e-nose in Indonesia, where the prevalence of TB is still high in the community (7), so that a portable, easy-to-use, and inexpensive screening tool will be helpful. In particular, this study aims to investigate the potential of an e-nose as a screening tool compared to the screening with clinical symptoms and chest radiology, which are currently used as a standard. Further, we aim to analyze the time and cost of a screening algorithm with e-nose to obtain additional detection of TB cases; and reducing the gap in TB case detection in Indonesia.

The urgency of research lies in: developing technology as a solution to increase the effectiveness of priority disease programs (tuberculosis), improve active TB case detection to improve the quality of health services, and increasing standards for TB screening. The main targets of this research are residents of the municipality of Yogyakarta and Kulon Progo District, Yogyakarta Special Region Province. The municipality of Yogyakarta was chosen because it represents an area with a high TB prevalence and Kulon Progo District was chosen because it represents a region with many remote areas with difficult access to the health center, thus the e-nose. The municipality of Yogyakarta has 18 Puskesmas (primary health centers) and 21 hospitals (8), while Kulon Progo District has 21 Puskesmas (primary health centers) and nine hospitals (9). The estimated incidence of TB in 2019 in the municipality of Yogyakarta is 1,400 cases, and in Kulon Progo district is 1,033 cases (10). In 2017, two-thirds of TB cases were detected in the municipality of Yogyakarta (10). In addition, Surakarta City is also used as a study site because in 2018, the detection of TB cases in Surakarta decreased by 1,651 compared to 2017 (11). The Surakarta city has 17 Puskesmas, 14 general hospitals, and 4 specialist hospitals (12).

## 1.2. Research Objectives

1.2.1. Investigate the potency of an e-nose as a screening tool.

1.2.2. Analyze the time and cost of a screening algorithm with e-nose to obtain additional detection of one TB case.

# CHAPTER 2. RESEARCH METHODOLOGY

## 2.1. Research Design

Before the e-nose is used as a screening tool, the e-nose was trained to recognize the breathing patterns. In the e-nose training phase, a case-control study was conducted, involving 27 TB patients taken from Respira Lung Hospital, Yogyakarta, and 24 healthy people as controls were taken from the environment around TB patients, to represent a population with the same socio-economic conditions. As part of the routine examination, all participants are characterized by clinical symptoms (*e.g.,* persistent cough, unintentional ≥5% weight loss, and night sweats), CXR, smear microscopic, Xpert MTB/Rif examination. For study purposes, we added sputum culture, HIV test, and e-nose breath test.

In the first phase or validation phase, a cross-sectional study will be carried out to validate the e-nose device (validation phase). The research will be conducted on 395 presumptive TB patients in Surakarta General Hospital, Puskesmas in the municipality of Yogyakarta and Kulon Progo District. As in the training phase, all study participants in the validation phase are characterized by clinical symptoms (*e.g.,* persistent cough, unintentional > 5% weight loss, and night sweats), CXR, smear microscopy, Xpert MTB / Rif examination as a reference, and e-nose breath test through air-collecting bag.

In the second phase or screening phase, cross-sectional research will be conducted in the municipality of Yogyakarta and Kulon Progo District, which will involve 1,383 respondents (screening phase). Along with the launch of an active TB case finding activity, e-nose will be paired with symptom screening and the use of chest radiology (CXR) in this activity. The mobile clinic team consisting of doctors, radiology officers, laboratory workers, and nurses will travel to the area with high TB risk, i.e., the waiting rooms of an outpatient clinic in primary health centers and hospitals, slums, boarding houses, boarding schools, and prisons.

The study participants will be asked to breathe normally through an air collecting bag until the collecting bag is full and will be asked whether they have TB symptoms, especially main symptoms of TB (cough with sputum> 2 weeks, night sweats without activity, unintended weight loss) and other symptoms of TB (cough with blood, intermittent fever > 1 month, enlargement of lymph nodes, shortness of breath and chest pain), and undergo a chest radiological examination.

Patients with positive radiological results and/ or breath tests and/ or coughing for more than two weeks or cough with blood or having extrapulmonary TB symptoms will be referred for confirmation of the diagnosis using the tests recommended by WHO, which have a higher specificity, namely Xpert MTB / Rif. The breath test performance as a TB screening test will be compared with the performance of symptoms and/ or chest radiology. The Xpert MTB / Rif is used as a reference because of its widespread use, minimizes sputum mobility, and provides fast results (within 2 hours). Participants' clinical and demographic data will be collected. The time and cost of an e-nose screening algorithm for additional detection of 1 TB case will be analyzed.

**Figure 1**. **Flowchart of screening with the e-nose, clinical symptoms, and chest radiography**

Mobile clinic for TB active case finding goes to the waiting rooms of outpatient clinic in primary health centres, hospitals, slums, boarding houses, boarding schools, and prisons. All participants are screened with portable CXR, e-nose, and main clinical symptoms

Participants with breath test (-) AND cough >2 weeks or coughing up blood (-) AND extrapulmonary TB symptoms (-) AND chest radiography (-)

Participants with abnormal CXR OR breath test (+) OR cough >2 weeks or coughing up blood (+) OR extrapulmonary TB (+) symptoms (+)

Refer for Xpert MTB/Rif examination for confirmation of diagnosis

- Analysis of the performance of breath test in comparison to symptoms or chest radiography screenings
- Time and cost analysis of breath test as a TB screening test

## 2.2. Research Population

### 2.2.1. Population

The population in the validation phase is children and adults who come to the Surakarta General Hospital and primary health centers in Yogyakarta and Kulon Progo District. In the screening phase, the population is children and adult residents in the municipality of Yogyakarta and Kulon Progo District, Yogyakarta Province.

### 2.2.2. Sample

- e-Nose training machine

The training phase was carried out to 27 TB patients and 24 healthy people as negative controls. Based on Bruins *et al.* and Zetola *et al.* (11,12), this sample size can be accepted by the e-nose machine to recognize the breathing pattern.

- Validation phase

The minimum sample required for the validation phase is calculated using a formula n=$\frac{{Z_{\propto/2}}^{2}xSNx(1-SN)}{d^{2}}/P$ (13)

with $Z_{\propto/2}$ = 1.96, SN value of 90%, d value of 5%, P value of 35%, thus the minimum number of respondents needed is 395 respondents.

- Screening phase

The number of samples in the screening phase is calculated with the same formula as the validation phase with $Z_{\propto/2}$1.96, SN value of 90%, d value of 5%, P value of 10%; thus the minimum number of respondents needed is 1.383 respondents.

### 2.2.3. Inclusion Criteria

Validation Phase:

- Adult and children
- Agree to participate in the study (sign *informed consent*)*.*
- Able to produce a sample for Xpert MTB/Rif examination
- Able to produce exhaled air sample

Screening phase:

- Adult and children
- Live in the municipality of Yogyakarta and Kulon Progo district.
- Agree to participate in the study (sign *informed consent*)*.*
- Currently not in TB treatment.

### 2.2.4. Exclusion Criteria

- Unable to breathe normally for two minutes due to respiratory illness
- Incomplete data of supporting examination
- Invalid measurements of breath tests

## 2.3. Blinding

To ensure the validity, the study uses triple-blind masking, in which the research subjects, breath sample takers, and laboratory sample examiners do not know the results of each sampling that has been done. The final data processor is also blinded to the results of Xpert or other supporting examinations. The breath sampling data are saved in graphic form, of which interpretation will be done later by the data processor at the final stage.

##

## 2.4. Tools and Materials

The e-nose system consists of a sampling system (1-3), a sensor array system (4), and a data acquisition system (5 and 6):


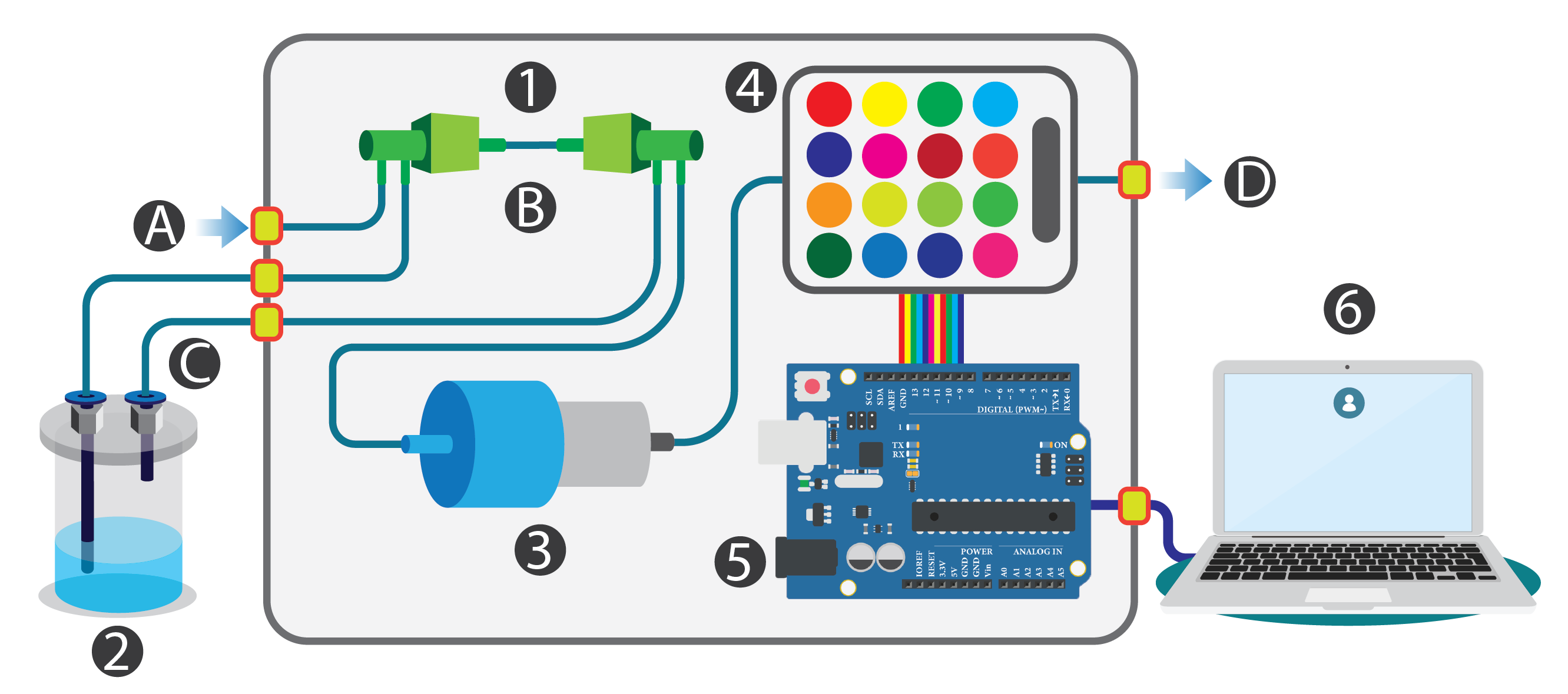


The e-nose software system consists of two programming parts: microcontroller programming (Arduino MEGA 2560) and data logger software programming (Windows). Participants breath normally through disposable air collecting bag until the collecting bag is full. Air collecting bag connected to HEPA-filter to protect electronic-nose contaminated with bacteria and virus. To prevent transmission of COVID-19, we take several precautions, such as N95 masks, surgical masks, face shields, hand schoons, hand sanitizers, 70% alcohol, and medical waste boxes.

## 2.5. Research Procedure

### 2.5.1. Collecting of the exhaled breath sample

After the doctor confirms the patient meets the inclusion and exclusion criteria, the DOTS officer will explain the study and ask for informed consent. The officer provides an air collecting bag to collect breath samples by closing the oxygen tube. The officer will give directions for taking breath samples into the air collecting bag. The officer will still accompany the patient when taking breath samples in a room designated for collecting sputum within a 1-meter distance from the patient. Participants will be asked to take a deep breath while still wearing a mask two times. During the third breath, participants are asked to take a deep breath and exhale (forced expiratory volume) into a single-use air collection bag until the collecting bag is full then the top of the collecting bag is tied. After the breath collecting bag is full and tied, the bag is put into a closed box and handed over to the officer. The participants will take breath for 2 times.

The full air collecting bag is then connected to the HEPA-filter with the connectors on one side and the other side connected to the e-nose machine. After using the e-nose, the officer will disinfect the equipment, i.e., the e-nose machine, connecting hose, and surrounding objects using 70% alcohol, then wait until the alcohol dry and the e-nose is ready to use for the next participant. The used air collecting bags, connectors, and HEPA-filters are then put into medical waste boxes.

The officer of health worker who operates the e-nose machine and takes the breath samples will receive training regarding the research outline, how to operate the e-nose machine and training in using the personal protective equipment (PPE) according to the protocol given by the researcher. The protocol that will be carried out in anticipation of COVID-19 transmission from sampling is as follows:

1. The e-nose machine will not be removed from the lab room until finished and will be wrapped in plastic wrap
2. If the machine can be connected to an internet connection, the data will be accessed electronically using the internet network, and the machine does not need to be removed.
3. If the researcher is required to enter the lab room, the researcher must follow the applicable health protocol and use the PPE according to medical procedures
4. The air collecting bag is specially designed for taking breath samples and is single-use, after completion it will be disposed of in infectious medical waste according to health protocols
5. The filled air collecting bag is tied and put in an airtight closed box to minimize transmission before the sample bag is connected to the e-nose machine.

The side effects of taking three times forced expiratory volume breath samples are generally rare. Studies report that the most common side effects are discomfort in the form of nausea, vomiting, and lightheadedness when done more than ten times in a row. Therefore, the anticipation of this study is to take a maximum of 3 breaths per time. If side effects occur, the nurse has been trained to assist by positioning the patient in a Trendelenburg position where the patient is put in a lay position, and the legs are elevated, then the patient is given additional oxygen supplementation with a nasal cannula. Evaluation of side effects will be conducted again after 15 minutes later and reported to the responsible doctor and researcher.

### 2.5.2. Risk Control and Mitigation of SARS-CoV Infection Transmission

In order to prevent the transmission of COVID-19 during the study, researchers have made working procedures that apply health protocols and use of PPE following the 5th revision of COVID-19 prevention and control guidelines by the Ministry of Health and the practical instructions and standard operating procedure of Surakarta General Hospital and primary health centers.

The process of informing the study to the participants, filling informed consent, and the Case Report Form (CRF) were carried out by trained officers using PPE level 2 (surgical masks, gowns, gloves, face shields/goggle). The accompanying staff for collecting participant breath samples and officers who operate the e-nose machine using PPE standard level 3 (N95 masks covered with surgical masks, gowns, face shields/goggles, rubber surgical gloves, head covers, protective shoes).

Medical waste such as breath sample collection bags, connectors, HEPA-filters, tissue/alcohol cotton, rubber surgical gloves, surgical masks/N95 is put into the trash can for infectious medical waste categories. Henceforth, the decontamination and destruction process is given to the infection prevention section and the medical waste treatment protocol at the Surakarta General Hospital and primary health centers.

### 2.5.3. Collection of patient clinical and demographic data

Age, weight, height, sex, smoking habits, alcohol consumption, comorbidities (diabetes, asthma, CPOD, HIV, flu, bronchitis, bronchiectasis, lung fibrosis, lung abscess, empyema, polycystic lung disease), comedication (inhaled drugs and antibiotics), occupation, food and drink consumed before the breath test. Two researchers will double-enter all data into a database and ensure no missing data or typing errors.

### 2.5.4. Analysis

**e-Nose data interpretation**

Multivariate (chemometric) data analysis uses the open-source programming language R version 3.5.1 and Python version 3.7. The R programming language is a programming language intended for statistical computation (such as linear and nonlinear modeling, classical statistical tests, time-series analysis, classification, and clustering) and graphics processing. R is used as a data processing machine consisting of a feature extraction function and a multivariate data analysis function. The GUI for multivariate data analysis software is built using Microsoft Visual Studio 2019.

The gas sensor response profile during a data collection cycle consisting of a delay phase, a sampling phase, and a purging phase. For each data collection, large data are obtained, and possibly many redundant data (10 data arrays per second); thus, the feature extraction procedure is applied to obtain important information from a sensor response. In this study, several feature extraction methods are used and compared to obtain the best accuracy results. For the e-nose data, the feature extraction method is conducted by taking the average value of each sensor's sampling data.

The program will perform a feature extraction procedure for all data files (* .csv format) in the selected folder using the RUN command. The feature extraction results will be automatically saved in the "results" folder. We use 16 feature extraction methods, where V*_i,j_* is the *i*-data (10 data per second of data collection) for the *j*-sensor. In the R programming, PCA analysis is performed with a "prcomp" function in the library base package, while t-SNE analysis is performed with the "Rtsne" function in the Rtsne library package. LDA is used to see the distribution of classified data using the supervised learning LDA model. Data with high dimensions will be transformed into low dimensional data using the linear discrimination analysis method. In the case of classification of two class labels, one discriminant function is obtained. The LDA procedure in this study uses the "lda" function in the MASS library package. Simulated Annealing is used as a feature selection method, looking for the most important features and removing redundant features, using an algorithm that mimics how the metal cooling process works. Simulated Annealing in R language is combined with LDA as a learning model through the subselect library package*.*

Classification and Regression are the main programs in chemometric data analysis. Both models are built using the library caret package (Classification and Regression Training), which provides important chemometric data analysis functions, such as data splitting, pre-processing, feature selection, training, and tuning models and variable importance estimation. A receiver operating characteristic (ROC) is used to perform analysis of 2 label classes. The caret library is also used in the ROC analysis procedure. A radar plot is used to display the variability of the average data for each label class aiming to be the initial hypothesis that there are differences in sensor responses to different label classes.

The Python programming language is also used in chemometric data analysis procedures. Python is a flexible programming language for various computational purposes. Chemometric data analysis procedures in the Python environment require several support packages, such as Numpy, Pandas, Sklearn, TensorFlow, and Keras*.* Anaconda is used in this research as a data science platform that provides a machine learning library and works on Python's latest version. In this study, TORCLIB.py is developed and built with various libraries, especially for chemometric data analysis.

**Statistical Analysis**

In the validation phase, we will calculate the sensitivity, specificity, positive predictive value (PPV), and negative predictive value (NPV) of the breath test using the Xpert MTB/Rif as the reference standard. In each variable (such as age, body mass index), one stratum’s Receiver Operating Characteristic (ROC)-curve indicating the breath test's sensitivity and specificity is compared with another stratum’s ROC-curve. An association between the breath test's variable and sensitivity-specificity is indicated by a significant difference of an AUC between strata (*p* < 0.05).

In the screening phase, the performance of screening with a breath test will be compared with screening with clinical symptoms or CXR examination by calculating positive and negative agreements between the breath test and clinical symptoms or CXR examination. The time and cost of a screening algorithm with e-nose to obtain additional detection of one TB case will be calculated as the mean of time needed and mean of cost spent from the beginning of screening with the e-nose until the detection of the case. Statistical analysis is performed using STATA/SE 15 (License: Universitas Gadjah Mada).

**CHAPTER 3. ETHICAL CONSIDERATIONS**

The research will be conducted following the Helsinki Declaration of 2013 and Good Clinical Practice principles, and with the approval of the Ethics Committee, Faculty of Medicine, Public Health and Nursing, Gadjah Mada University, Yogyakarta, Indonesia. The pilot machine learning study (training phase) was approved by the ethics committee with the number KE / FK / 0769 / EC / 2019 issued on July 5, 2019, which was valid for one year.

In the validation phase, the doctor in charge of the patient at the Surakarta General Hospital and primary health centers will provide information to presumptive TB patients about the study and offer the patient to meet with one of the research teams or officers. They are trained to provide information about the research. The research team or officers will provide information about this research verbally and in writing. If the patient agrees, the patient will sign an informed consent. If the patient is under 12 years of age, informed consent is obtained from the parents. For adolescents aged 12-18 years, an informed accent will be requested where the child also gives consent for participating in the study.

In the screening phase, nurses/officers in the mobile clinic team will provide information to potential participants about this study. If the subjects agree, the research subjects will sign an informed consent.

Participants will be given an explanation regarding the possible side effects of the breath sampling process if it is done excessively, such as the occurrence of vagal reflexes in the form of a sensation of dizziness, onset, spinning. If this happens, the participant will be positioned into the Trendelenburg position with the feet in a more elevated position from the head. Participants will not be paid to contribute to this research, but if there are costs incurred by participants for participating in this research, these costs will be exchanged.

The research team will keep all data safe, and only the researchers have access to this data. Data can be disclosed to legal authorities and monitoring and auditing officers. In this study, no sample will be taken abroad and used for any equipment development abroad.

A doctor who is not involved in the research (independent physician) is provided to answer potential participants' questions if they need it. There is one responsible doctor in each hospital/study site who is involved in this study.

**CHAPTER 4. RESEARCH TIMELINE**

Validation phase

| No | Activities | Month | | | | | | | |
| --- | --- | --- | --- | --- | --- | --- | --- | --- | --- |
|  |  | 1 (Sept. 2020) | 2  (Oct. 2020) | 3 (Nov. 2020) | 4 (Dec. 2020) | 5 (Jan. 2021) | 6 (Feb. 2021) | 7 (March 2021) | 8 (April 2021) |
| 1. | Ethical clearance | x | x |  |  |  |  |  |  |
| 2. | Explanation of the research to Surakarta General Hospital, , and primary health centers of Yogyakarta and Kulon Progo district | x | x |  |  |  |  |  |  |
| 3. | Training in using electronic-nose and simulation of research flow |  | x |  |  |  |  |  |  |
| 4. | Patient recruitment for the electronic-nose training phase |  |  | x | x | x | x | x | x |
| 5. | Data analysis |  |  |  |  |  |  |  |  |
| 6. | Manuscript writing and publication | x | x | x |  |  |  |  |  |

Screening phase

| No | Activities | Month | | | | | | | | | | | |
| --- | --- | --- | --- | --- | --- | --- | --- | --- | --- | --- | --- | --- | --- |
|  |  | 1 | 2 | 3 | 4 | 5 | 6 | 7 | 8 | 9 | 10 | 11 | 12 |
| 1. | Explanation of the research to the District Health Offices, and primary health centers in Yogyakarta and Kulon Progo district | x |  |  |  |  |  |  |  |  |  |  |  |
| 2. | Training in using electronic-nose and simulation of research flow |  | x |  |  |  |  |  |  |  |  |  |  |
| 3. | Electronic-nose TB screening |  |  | x | x | x | x | x | x | x |  |  |  |
| 4. | Data analysis |  |  |  |  |  |  |  |  |  | x |  |  |
| 5. | Manuscript writing and publication |  |  |  |  |  |  |  |  |  |  | x | x |

# REFERENCES

1. World Health Organization. Global Tuberculosis Report 2019 [Internet]. Geneva; [cited 2019 Nov 27]. Available from: https://apps.who.int/iris/bitstream/handle/10665/329368/9789241565714-eng.pdf

2. World Health Organization. Systematic Screening for Active Tuberculosis: Principles and Recommendations. Geneva; 2015. Available from: https://www.who.int/tb/tbscreening/en/

3. van’t Hoog AH, Langendam MW, Mitchell E, Cobelens FG, Sinclair D, Leeflang MMG, et al. A systematic review of the sensitivity and specificity of symptom- and chest-radiography screening for active pulmonary tuberculosis in HIV-negative persons and persons with unknown HIV status. In: World Health Organization, editor. WHO/HTM/TB. Geneva: World Health Organization; 2013. p. 44–7. (Systematic screening for active tuberculosis: principles and recommendations).

4. Fens N, Zwinderman AH, van der Schee MP, de Nijs SB, Dijkers E, Roldaan AC, et al. Exhaled breath profiling enables discrimination of chronic obstructive pulmonary disease and asthma. Am J Respir Crit Care Med. 2009 Dec;180(11):1076–82.

5. Dragonieri S, Annema JT, Schot R, van der Schee MP, Spanevello A, Carratu P, et al. An electronic nose in the discrimination of patients with non-small cell lung cancer and COPD. Lung Cancer. 2009 May;64(2):166–70.

6. Riono P. TB Elimination in Indonesia [Internet]. 2018 [cited 2018 Nov 13]. Available from: http://www.depkes.go.id/resources/download/info-terkini/materi pra rakerkesnas 2018/Pakar TBC.pdf

7. World Health Organization. Health System Profile of Indonesia [Internet]. 2013 [cited 2016 Mar 20]. Available from: http://www.ino.searo.who.int/en/Section3_24.htm

8. Dinas Kesehatan Kota Yogyakarta. Profil Kesehatan Tahun 2019 Kota Yogyakarta. Yogyakarta; 2020. Available from: https://kesehatan.jogjakota.go.id/uploads/dokumen/profil_dinkes_2019_data_2018.pdf

9. Dinas Kesehatan Kabupaten Kulon Progo. Profil Kesehatan Tahun 2020. Yogyakarta; 2020. Available from: https://dinkes.kulonprogokab.go.id/detil/726/profil-kesehatan

10. Dinas Kesehatan Daerah Istimewa Yogyakarta. Profil Kesehatan Daerah Istimewa Yogyakarta Tahun 2019. Yogyakarta; 2020. Available from: https://www.dinkes.jogjaprov.go.id

11. Kementerian Kesehatan Republik Indonesia. Peran Pemerintah Daerah dalam Menurunkan Prevalensi Tuberkulosis Kota Surakarta [Internet]. Jakarta; 2019 [cited 2020 Oct 1]. Available from: https://www.kemkes.go.id/resources/download/info-terkini/rakerkesnas-2019/SESI II/Kelompok 5/2-Peran-Pemerintah-Daerah-dalam-Menurunkan-Prevalensi-TB.pdf

12. Badan Pusat Statistik. Badan Pusat Statistik Kota Surakarta [Internet]. 2020 [cited 2020 Oct 1]. Available from: https://surakartakota.bps.go.id/statictable/2020/04/01/178/jumlah-rumah-sakit-umum-rumah-sakit-khusus-rumah-sakit-bersalin-rumah-bersalin-puskesmas-klinik-balai-kesehatan-posyandu-dan-polindes-menurut-kecamatan-di-kota-surakarta-2018-dan-2019.html

13. Bruins M, Rahim Z, Bos A, Van De Sande WWJ, Endtz HP, Van Belkum A. Diagnosis of active tuberculosis by e-nose analysis of exhaled air. Tuberculosis [Internet]. 2013 Mar [cited 2020 Oct 26];93(2):232–8. Available from: https://pubmed.ncbi.nlm.nih.gov/23127779/

14. Zetola NM, Modongo C, Matsiri O, Tamuhla T, Mbongwe B, Matlhagela K, et al. Diagnosis of pulmonary tuberculosis and assessment of treatment response through analyses of volatile compound patterns in exhaled breath samples. J Infect [Internet]. 2017 Apr 1 [cited 2020 Oct 26];74(4):367–76. Available from: https://pubmed.ncbi.nlm.nih.gov/28017825/

15. Fenn Buderer NM. Statistical methodology: I. Incorporating the prevalence of disease into the sample size calculation for sensitivity and specificity. Acad Emerg Med [Internet]. 1996 [cited 2020 Oct 26];3(9):895–900. Available from: https://pubmed.ncbi.nlm.nih.gov/8870764/
