# Supplementary material for: eNose-TB: A trial study protocol of electronic nose for tuberculosis screening in Indonesia
Source: PLoS One. 2021 Apr 21;16(4):e0249689. doi: 10.1371/journal.pone.0249689 (PMC8059810; doi:10.1371/journal.pone.0249689)
Supplement: S1 Appendix — (PDF) [file pone.0249689.s004.pdf]

PANDUAN WAWANCARA

1. Data pribadi

(Tanyakan ke pasien atau pengantar pasien)

a. Tanggal pengambilan data : _ _-_ _-_ _ _ _ *(tuliskan tanggal-bulan-tahun)*

b. Kode pasien : _ -_ _ _ _ *(tuliskan sesuai kode yang dicantumkan pada form inklusi-eksklusi)*

c. Jenis kelamin : L / P *(tuliskan jenis kelamin sesuai KTP atau akta lahir)*

d. Tanggal lahir : _ _-_ _-_ _ _ _ *(tuliskan tanggal-bulan-tahun)*

e. Alamat : ……………………………………………………………….

(tuliskan alamat tempat tinggal sekarang, bukan alamat di KTP)

f. Nomor telepon : ………………………………………… *(tuliskan nomor telepon yang dapat dihubungi)*

g. Indeks Massa Tubuh : TB ………… cm; BB …………….. kg

h. Pekerjaan : …………………………………………

i. Status Merokok

☐ Ya

Jika Ya, Kapan terakhir merokok ? ……………………………………………………

1. Tidak pernah atau ≥8 jam yang lalu
2. <8 jam yang lalu

(lingkari jawaban yang sesuai)

☐ Tidak

j. Pernah minum/konsumsi alkohol

☐ Ya

Jika Ya, Kapan terakhir mengkonsumsi alkohol ? ………………………………………..

1. Tidak pernah atau ≥8 jam yang lalu
2. <8 jam yang lalu

(lingkari jawaban yang sesuai)

☐ Tidak

k. Konsumsi Makanan dan Minuman Sebelum Tes Nafas

a. Kapan terakhir makan ?

1. Tidak makan atau makan sebelum tes nafas ≥ 1 jam yang lalu

2. Makan sebelum tes nafas <1 jam yang lalu

(lingkari jawaban yang sesuai)

b. Makan apa ?

(tulis dengan lengkap)

…………………………………………………………………………………...

c. Kapan terakhir minum ?

1. Tidak minum atau minum sebelum tes nafas ≥ 1 jam yang lalu

2. Minum sebelum tes nafas <1 jam yang lalu

(lingkari jawaban yang sesuai)

d. Minum apa ?

(tulis dengan lengkap)

……………………………………………………………………………………

Kategori makanan dan minuman :

1. Daging unggas
2. Kopi
3. Teh
4. Susu
5. Lainnya

(lingkari jawaban yang sesuai)

2. Penyakit Penyerta dan Ko-medikasi

(Centang yang sesuai)

1. Penyakit Penyerta
   - ☐ HIV/AIDS
   - ☐ Diabetes
   - ☐ PPOK (penyakit paru obstruktif kronis)
   - ☐ Asma
   - ☐ Bronkitis
   - ☐ Bronkiektasis
   - ☐ Fibrosis paru
   - ☐ Abses paru
   - ☐ Empiema
   - ☐ Penyakit paru polikistik
   - ☐ Flu
2. Ko-medikasi

- ARV/obat anti-retroviral (sebutkan nama obat dan dosisnya)
  …………………………………………………………………….
- Obat inhalasi untuk asma (sebutkan nama obat dan dosisnya)
  …………………………………………………………………….
- Obat inhalasi untuk PPOK (sebutkan nama obat dan dosisnya)
  …………………………………………………………………….
- Antibiotik (sebutkan nama obat dan dosisnya)
  …………………………………………………………………….
- Lainnya (sebutkan nama obat dan dosisnya)
  …………………………………………………………………….

INTERVIEW GUIDE

1. Personal Data

(Ask to patients or their legal guardians)

a. Date : _ _-_ _-_ _ _ _ *(write the date-month-year)*

b. Patient code : _ -_ _ _ _ *(write the code according to the code written in the inclusion-exclusion form)*

c. Sex : M/ F *(write the sex according to what is written in the national identity card or birth certificate)*

d. Birth date : _ _-_ _-_ _ _ _ *(write the date-month-year)*

e. Address : ....

(write the current address, not the address written in the national identity card)

f. Phone number : .... *(write the phone number that can be contacted)*

g. Body Mass Index : Height .... cm; Weight .... kg

h. Occupation : ....

i. Smoking habits :

☐ Yes

If Yes, when was the last time you smoked?

1. never or ≥8 hours ago
2. <8 hours ago

(circle the appropriate answer)

☐ No

j. Alcohol consumption

☐ Yes

If Yes, when was the last time you consumed alcohol?

1. never or ≥8 hours ago
2. <8 hours ago

(circle the appropriate answer)

☐ No

k. Food and beverage consumption before the breath test

When did you last take food?

1. not having prior intake or having prior intake ≥1 hour ago

2. having prior intake <1 hour ago

(circle the appropriate answer)

What kind of food did you consume?

(write in full sentences)

……………………………………………………………………………...

When did you last take beverages?

1. not having prior intake or having prior intake ≥1 hour ago

2. having prior intake <1 hour ago

(circle the appropriate answer)

What kind of drink did you consume?

(write in full sentences)

………………………………………………………………………………

The category of food and beverages:

1. Poultry meat

2. Coffee

3. Tea

4. Milk

5. Others

(circle the appropriate answer)

2. Comorbidities and Co-medication

(Tick the appropriate answer)

1. Comorbidities
   - ☐ HIV/AIDS
   - ☐ Diabetes
   - ☐ Chronic Obstructive Pulmonary Disease/COPD
   - ☐ Asthma
   - ☐ Bronchitis
   - ☐ Bronchiectasis
   - ☐ Lung fibrosis
   - ☐ Lung abscess
   - ☐ Empyema
   - ☐ Polycystic lung disease
   - ☐ Flu

2. Co-medication
    - ARV/anti-retroviral drugs (mention the name of drugs and the dose)
    …………………………………………………………………….
    - Inhalation drugs for asthma (mention the name of drugs and the dose)
    …………………………………………………………………….
    - Inhalation drugs for COPD (mention the name of drugs and the dose)
    …………………………………………………………………….
    - Antibiotic (mention the name of drugs and the dose)
    …………………………………………………………………….
    - Others (mention the name of drugs and the dose)
    …………………………………………………………………….
