# Supplementary material for: eNose-TB: A trial study protocol of electronic nose for tuberculosis screening in Indonesia
Source: PLoS One. 2021 Apr 21;16(4):e0249689. doi: 10.1371/journal.pone.0249689 (PMC8059810; doi:10.1371/journal.pone.0249689)
Supplement: Attachment — Submitted filename: Response to Reviewers.docx [file pone.0249689.s005.docx]

**Response to reviewers’ comments PONE-D-20-33305**

The authors would like to thank the reviewers for their careful reading and their valuable comments. Below please kindly find the reviewers’ comments addressed on a point-to-point basis.

Comments to the Authors:
Reviewer #1: This is an excellent plan addressing an important question, and making use of a plan in progress to thrive for a zero-TB city of Yogyakarta. The plan is carefully designed, with a pre-specified sample size to develop and validate the test; and next, to sue is for screening for TB.

1) If I understand it correctly, the measurement of breath samples collected will be processed by e-nose on the spot; if that is correct, how will the safety precautions to prevent the transmission of TB and Covid-19 be secured? will all sites have a dedicated room for e-nose measurements?

Response: We thank the reviewer for this comment. All sites will have a dedicated room for eNose-TB measurements, *i.e.*, a particular isolation room. The officer who operates the eNose-TB device uses level 3 personal protective equipment (N95 mask, gloves, goggles, gown, and boots or closed shoes). We also use disposable equipment (HEPA-filter, connector, and air collecting bag), apply disinfectant procedure after each breath sampling, and ensure that the physical distancing measurement is applied during the sampling collection. These safety precautions can be found in the lines 302-308, and 454-460 (clean version of the manuscript)

2) the protocol aims at enrolling 40 children; will there be a lower limit in age, e.g., 10 years and over, to make sure that the child-participants have adult-phenotype pulmonary TB and that these young participants can follow the instructions for breath collection, and produce sputum for GeneXpert?

Response: We kindly thank the reviewer for his/her careful reading. The lower limit in age for children will be 4 years old, as the child-participants in this age and over are considered able to follow the instructions for breath collection. In case the child-participants cannot produce sputum for GeneXpert, they will be asked to collect their stool samples for the Xpert examination, as this method has been shown as reliable in diagnosing TB (DOI: 10.1128/JCM.02057-18). We added this information in the lines 342-346 (clean version of the manuscript). We aim to include young children (≥ 4 years old) to cover various pulmonary TB phenotypes.

3) who is the sponsor of the study?

Response: We thank the reviewer for this query. The sponsor of the study is the Ministry of Research, Technology, and Higher Education of Republic Indonesia. The sponsor had no role in the study design, data collection and analysis, decision to publish, or preparation of the manuscript. We wrote it down in the study protocol (page 1). This information will also appear in the published paper; however, this information could not be put in this manuscript draft in accordance with the PloS One guideline.

4) who is the manufacturer of the e-nose device, and the software?

Response: We kindly thank the reviewer for this query. The manufacturer of the eNose-TB device is the Department of Physics, Faculty of Mathematics and Natural Sciences, Universitas Gadjah Mada, Indonesia. The software was developed and validated by the same department. We have added this information in the lines 238-240 (clean version of manuscript).

5) you plan to complete data collection by December, 2012; what if Covid-19 jeopardizes your plans?

Response: We thank the reviewer for noticing this. We will put our best effort into finishing the study in the planned time frame. However, if COVID-19 jeopardizes our plans, we will extend the study until a maximum of another year and add to the number of study sites. We added this information in the lines 308-310 (clean version of manuscript).

textual suggestions:

page 4, line 82: due to infections from TB: change into 'due to TB'

page 8, line 172: unable to: change into inability to . . 

page 13, line 287: Afterward,  . . change into: Next,  .. 

line 292: As the anticipation of Covid transmission . . . considering the risks of TB and Covid-19 transmission, . .

Response: We thank the reviewer for these inputs. We changed the text according to the suggestions (page 4 line 82, page 8 line 171, page 13 line 297, page 13 lines 302-303, clean version of manuscript).

page 14, line 303: smoking habits; my impression was that you plan to exclude smokers; please explain.

Response: In our previous pilot machine learning study, we excluded smokers. However, in this current study, we include smokers, and thus, we record their smoking habits to analyse the effect of smoking on the breath test results.

line 310: 'when was last time you smoked' (I guess you will be asking questions in local languages or Bahasa Indonesia; when was the last time . .(add 'the')

Response: We kindly thank the reviewer for recommending this. We have added “the” to the sentences (page 14 line 324, clean version of manuscript). As highlighted by the reviewer, all questions will be asked in Bahasa Indonesia; we added this information in page 15 lines 331, clean version of the manuscript.

line 313: 'when did you last take food or beaverages'

Response: We kindly thank the reviewer for his/her recommendation. We changed the sentences as suggested (page 14 line 327, clean version of manuscript).

page 21, line 459; is it a vagal reflex, or perhaps more likely, a hyperventilation-induced response, caused by hypocarbia and respiratory alkalosis? I think that this is what should be anticipated; Trendelenburg positioning is not indicated then, and just a bit of rest, sitting or lying down a while will settle the case. This is much easier than Trendelenburg positioning which will also be more uncomfortable for participants of the study.

Response: We kindly thank the reviewer for recommending this. We have revised the sentences according to the reviewer’s suggestion (lines 410-413, clean version of manuscript).

Reviewer #2: The objectives are good. TB is a particular problem, especially amongst childrene and it i very difficult to separate from other symptoms to give the right interventions for control strategies. This manuscript is very confusing. It is all written in the present tense. It should be in the past tense as the study, I assume has already been done. It is very difficult to understand the initial study and then the validation study. Were these done in sequence or was the same set of data used for both??

Response: We kindly thank the reviewer for this comment. The initial study (previous pilot machine learning study) was conducted in the past. In contrast, the validation study and the screening study were not started when we submitted this manuscript. The validation study and the screening study are being conducted after the initial study, with a different study population and a different set of data.

We tried to point this out more clearly in the manuscript, as follows:

- ‘In a previous pilot machine learning study (unpublished), we trained the eNose-TB to recognize the breath pattern of TB patients and healthy controls.’ (lines 109-110, clean version of manuscript).
- ‘After the previous pilot machine learning study was finished, we aim to continue the study with a two-phase planned project, namely the validation and screening phases. The purpose of this current study is to validate the eNose-TB and investigate it as a screening tool for TB.’ (lines 160-163, clean version of manuscript).
- ‘After the validation phase, we will conduct a screening phase with a cross-sectional design.’ (lines 190-191, clean version of manuscript).

Most of the Figures are totally irrelevant to the objectives. Where are the PCS, where are the clustered dendograms or similar to demonstrate that it is possible to distinguis using their enose the TB vs no TB cohorts and the difference in VOC profiles?? Most of the Figures are about the software system and how the screen pages in the instrument look. These are irrelevant.

Response: We kindly thank the reviewer for his/her inputs. We revised the Figures by removing some images irrelevant to the study objectives, and including the following images:

**Fig 4.  The lab-made eNose-TB device connected to the sampling bag through a mouthpiece and HEPA filter**

**Fig 6.  The response of 16 sensors of eNose-TB for negative TB (a) and positive TB (b) subjects**

**Fig 7.  Change of temperature (a) and relative humidity (b) in the sensor chamber during the measurement**

We also added the following information:

Fig 4 and 5 show the eNose-TB system, which contains a sampling system (air collecting bag, HEPA-filter, valve, and micro-pump), a sensor array system, and a data acquisition system (Arduino and a personal computer). The micro-pump has a flow rate of about 1 milliliter per minute. This micro-pump functions to suck the breath sample into the gas sensor chamber and to push it out after the sensing process is complete. Meanwhile, HEPA filters are used to filter virus particles or other foreign objects other than VOCs from breath samples (lines 232-238, clean version of manuscript).

We use the Metal-Oxide-Semiconductor (MOS)-based gas sensors. All sensors used in the eNose-TB device are commercial sensors available in the market with a high level of replication (https://doi.org/10.3390/chemosensors7030029). There are 16 sensors used, with the characteristics of global selectivity. Based on the datasheet and our tests’ results, each sensor responds to at least 5 different gases, thus the detection range is at least 80 different VOC groups. With that coverage, the sensor array will form a relatively consistent pattern for each group of breath (lines 247-252, clean version of manuscript).

Fig 6 is a typical voltage response of 16 sensors in eNose-TB device to time. The sensor response begins with the baseline at the same level for 10 seconds, when the surrounding air is introduced into the gas sensor room. After that, the electronic valve will control that the breath sample is inserted into the gas sensor chamber for 120 s. The sensing process occurs after the 10th seconds to the 130th seconds, so the total sampling time is 120 seconds. When the sensing process is complete, the sensor room is cleaned with the ambient air until it returns to the original baseline level. To distinguish the breath response pattern between the negative and positive TB subjects, each sensor’s response during the sensing process is divided into 5 windows with an interval of 24 seconds each (lines 254-262, clean version of manuscript).

Furthermore, we extract the features from each window in the form of the median and standard deviation values with pre-processing in the form of standard and scaler. Each sensor has 10 characteristic values, which are then used as input into the machine learning model as a classification model, namely the support vector machine (SVM). The three main parameters of SVM for each set is a cost value of 10, with the kernel in the form of a linear function and using an optimizer in the form of a recursive feature elimination (RFE). Because the number of data in the pilot machine learning phase (training phase) was limited, we used a system performance validation method in the form of a leave-one-out cross-validation (LOO-CV) (lines 267-275, clean version of manuscript).

How good is the replication of the sensors used in this instrument? How many sensors are used?? What is the range of detection of different VOC groups. Are they complimentary or what?? None of this information is present.

Response: All sensors used in the eNose-TB device are commercial sensors available in the market with a high level of replication (https://doi.org/10.3390/chemosensors7030029). There are 16 sensors used, with the characteristics of global selectivity. Based on the datasheet and our tests’ results, each sensor responds to at least 5 different gases, thus the detection range is at least 80 different VOC groups. With that coverage, the sensor array will form a relatively consistent pattern for each group of breath. We added this information in the lines 247-252 (clean version of manuscript).

Were sputum samples taken and analysed to confirm TB or no TB patients?? Most of the relevant data around these questions are not included.

Response: We kindly thank the reviewer for his/her careful reading. To describe this data more clearly in the manuscript, we revised the text and added the following sentences:

- Participants’ sputum is taken and analyzed with Xpert MTB/Rif examinations to confirm the diagnosis of TB. An acid-fast-bacilli Ziehl-Neelsen microscopy is done as a part of the routine examination in the hospital. If a participant cannot produce sputum, he/she will be asked to collect a stool sample, as this method has been shown as reliable in diagnosing TB (DOI: 10.1128/JCM.02057-18) (validation phase section, lines 175-179, clean version of manuscript).
- Patients with positive radiological results or positive breath tests or coughing for more than two weeks or coughing up blood or showing extrapulmonary TB symptoms will be asked to collect their sputum or stool samples for Xpert MTB/Rif examinations to confirm the diagnosis of TB (screening phase section, lines 216-219, clean version of manuscript).

What about the sensitivity of metal oxide sensors to relative humidity. How was this overcome???

Response: We thank the reviewer for this query. The effects of temperature and humidity in the sensor room are measured in parallel (with a calibrated SHT31 sensor) during the sensing process. Fig 7 shows the change in temperature and relative humidity during the pilot machine learning study (training phase). This box plot is derived from all recorded data, summarized in the following table:

Changes in temperature and relative humidity during measurement were only about 0.5 C and 15%, respectively, which means that there is no significant effect on sensor response when referring to sensor characteristics in general (https://doi.org/10.3390/chemosensors7030029) (lines 277-282, clean version of manuscript).

The key scientific aspects are ignored while the process of the software in the instrument are focused on. Unfortunately, this does not help in understanding the Results. This manuscript needs to be completely rewritten in a format which is understandable and approaches the work systematically. I assume the work was all done and needs to be worded in the past tense. The work is interesting and would be useful but the present version is really not up to scratch and needs very major rewriting and presentation.

Response: We sincerely thank the reviewer for his/her comments and suggestions. We re-wrote the manuscript to present the study more clearly. We also simplify the explanation concerning the process of the software (from 23 lines in the previous version of the manuscript into 5 lines: lines 288-292, clean version of the current manuscript; and from 77 lines in the previous version of the manuscript into 10 lines: lines 363-372, clean version of the current manuscript). The validation and screening phases are written in a present tense or future tense as the validation phase is on-going, and the screening phase has not been conducted.
